# Identification of host dependency factors involved in SARS-CoV-2 replication organelle formation through proteomics and ultrastructural analysis

**DOI:** 10.1128/jvi.00878-23

**Published:** 2023-10-31

**Authors:** Felix Pahmeier, Teresa-Maria Lavacca, Sarah Goellner, Christopher J. Neufeldt, Vibhu Prasad, Berati Cerikan, Sreejith Rajasekharan, Giulia Mizzon, Uta Haselmann, Charlotta Funaya, Pietro Scaturro, Mirko Cortese, Ralf Bartenschlager

**Affiliations:** 1 Department of Infectious Diseases, Molecular Virology, Heidelberg University, Medical Faculty Heidelberg, Center for Integrative Infectious Disease Research, Heidelberg, Germany; 2 Systems Arbovirology, Leibniz Institute of Virology, Hamburg, Germany; 3 German Center for Infection Research, Heidelberg partner site, Heidelberg, Germany; 4 Electron Microscopy Core Facility, Heidelberg University, Heidelberg, Germany; 5 Division “Virus-Associated Carcinogenesis”, German Cancer Research Center (DKFZ), Heidelberg, Germany; The Peter Doherty Institute for Infection and Immunity, Melbourne, Victoria, Australia

**Keywords:** host-pathogen interactions, coronavirus, double-membrane vesicle

## Abstract

Positive-strand RNA viruses subvert the cellular endomembrane system for the generation of distinct compartments termed replication organelles (ROs) that harbor the site where viral RNAs are generated. In this study, we corroborate that the SARS-CoV-2 non-structural proteins 3 and 4 (nsp3 and nsp4) suffice to remodel the endoplasmic reticulum to form double-membrane vesicles similar to ROs observed in viral infection. Cellular membrane alterations induced by nsp3/4 expression were evaluated through electron tomography and confocal microscopy, and nsp3/4-associated host factors were identified using mass spectrometry. The role of these host factors in virus infection was determined using gene silencing, identifying several host proteins involved in the SARS-CoV-2 replication cycle. Combining the gene silencing approach with ultrastructural analysis of nsp3/4-expressing cells, we found that the host dependency factors FAM149B1, CCAR2, and ZC3HAV1 play a role in the formation of double-membrane vesicles in a replication-independent manner.

**IMPORTANCE:**

Remodeling of the cellular endomembrane system by viruses allows for efficient and coordinated replication of the viral genome in distinct subcellular compartments termed replication organelles. As a critical step in the viral life cycle, replication organelle formation is an attractive target for therapeutic intervention, but factors central to this process are only partially understood. In this study, we corroborate that two viral proteins, nsp3 and nsp4, are the major drivers of membrane remodeling in SARS-CoV-2 infection. We further report a number of host cell factors interacting with these viral proteins and supporting the viral replication cycle, some of them by contributing to the formation of the SARS-CoV-2 replication organelle.

## INTRODUCTION

The causative agent of COVID-19, severe acute respiratory syndrome coronavirus 2 (SARS-CoV-2), is an enveloped virus with a positive-sense single-strand RNA genome (+ssRNA) belonging to the Coronaviridae virus family. Coronaviruses have been associated with respiratory and intestinal infections ranging from common cold to multisystem inflammatory syndrome, pneumonia, and death (1). SARS-CoV-2 entry into target cells is initiated through interactions between the viral spike (S) protein located on the surface of virions and the receptor protein angiotensin-converting enzyme 2 (ACE2) residing on the cell surface. This association leads to proteolytic priming of S by transmembrane protease, serine 2 (TMPRSS2), exposing the S fusion peptide, which mediates fusion of host and virion membranes (2). Furthermore, in cells not expressing TMPRRS2, the virus can be endocytosed and primed by endosomal cathepsin L in a pH-dependent manner (3). After release of the approximately 30 kb +ssRNA genome into the cytosol, translation of the viral RNA occurs at the rough endoplasmic reticulum (ER). The first two open-reading frames (ORF) 1a and ORF1ab are translated as polyproteins and cleaved into the non-structural proteins (nsp) 1 to 16 by viral proteases (4 – 7). The papain-like protease PLpro located in nsp3 mediates the processing at the nsp1/2, nsp2/3, and nsp3/4 junctions, while the main protease 3CLpro in nsp5 cleaves the remaining junctions (7, 8). Expression of viral proteins during infection leads to the formation of a network of remodeled ER-derived membranes termed replication organelles (RO) consisting of double-membrane vesicles (DMVs), double-membrane spherules (DMSs), and convoluted membranes (CMs) (9 – 11). Replication intermediates including double-stranded and newly synthesized RNAs have been detected within DMVs, while the other virus-induced membranous elements lack this marker, suggesting that DMVs are the primary site of viral genome replication (12, 13).

The minimally required viral proteins for coronavirus DMV formation have been determined to be nsp3 and nsp4 (14 – 18), although other proteins such as nsp6 appear to be involved in other aspects of membrane remodeling during CoV infection, including nsp6’s function as a regulator of lipid transport (19). With an approximate mass of 220 kDa, nsp3 is the largest of the viral proteins and has been divided into several subdomains, which harbor enzymatic or structural functions (8, 20). The ubiquitin-like domains structurally resemble ubiquitin and have been demonstrated to bind ssRNA and the nucleocapsid protein N (20). The macrodomains 1 to 3 also have RNA-binding activity and are involved in hydrolysis of poly-adenosine diphosphate ribose (20). Importantly, inactivation of the protease activity in the PLpro domain located in nsp3 abrogated the formation of DMVs (15). Nsp4 is a relatively small viral protein that harbors no known enzymatic activity but serves a central structural function in membrane remodeling (14, 15).

In addition to viral factors, the host cell machinery is subverted by +ssRNA viruses for RO biogenesis. Examples to that include phosphoinositide-4-kinase III alpha exploited by the hepatitis C virus (HCV) (21) and, for the flaviviruses, the fatty acid synthase (22), the valosin-containing protein (23, 24), and the membrane shaping proteins of the atlastin and reticulon protein families (25, 26). For coronaviruses, inhibition of Golgi brefeldin A-resistant guanine nucleotide exchange factor 1 limited replication of the murine hepatitis virus (MHV) and altered virus-induced DMV morphology (27). Additionally, transmembrane protein 41B (TMEM41B) was demonstrated to be a pan-coronaviral host dependency factor that is involved in altering ER membrane lipid composition (28 – 30). Another study suggested that SARS-CoV DMV formation is closely linked to the ER-associated degradation tuning pathway and that the ER degradation enhancing alpha-mannosidase-like protein 1 (EDEM1) is present at, but not strictly required for, DMV formation (31). Recently, the signaling lipid phosphatidic acid (PA) was shown to be an important component of HCV and SARS-CoV-2 DMVs and that the pharmacological inhibition of the PA synthesis pathway impacts DMV morphology and virus replication (16). Furthermore, host factors involved in autophagosome formation, including the class III phosphatidylinositol 3 kinase (PI3K), were shown to contribute to DMV formation in HCV and SARS-CoV-2 (17). However, a more comprehensive list of host cell factors involved in the biogenesis of SARS-CoV-2 DMVs is lacking.

In this study, we investigated the role of both host and viral factors in SARS-CoV-2-induced DMV biogenesis. We employed a SARS-CoV-2 nsp3/4 expression system that induces the formation of DMV-like structures outside the context of viral replication and identified host cell factors involved in viral replication and DMV formation.

## RESULTS

### Characterization of SARS-CoV-2 nsp3 and nsp4 in context of single and polyprotein expression

**Fig 1:**
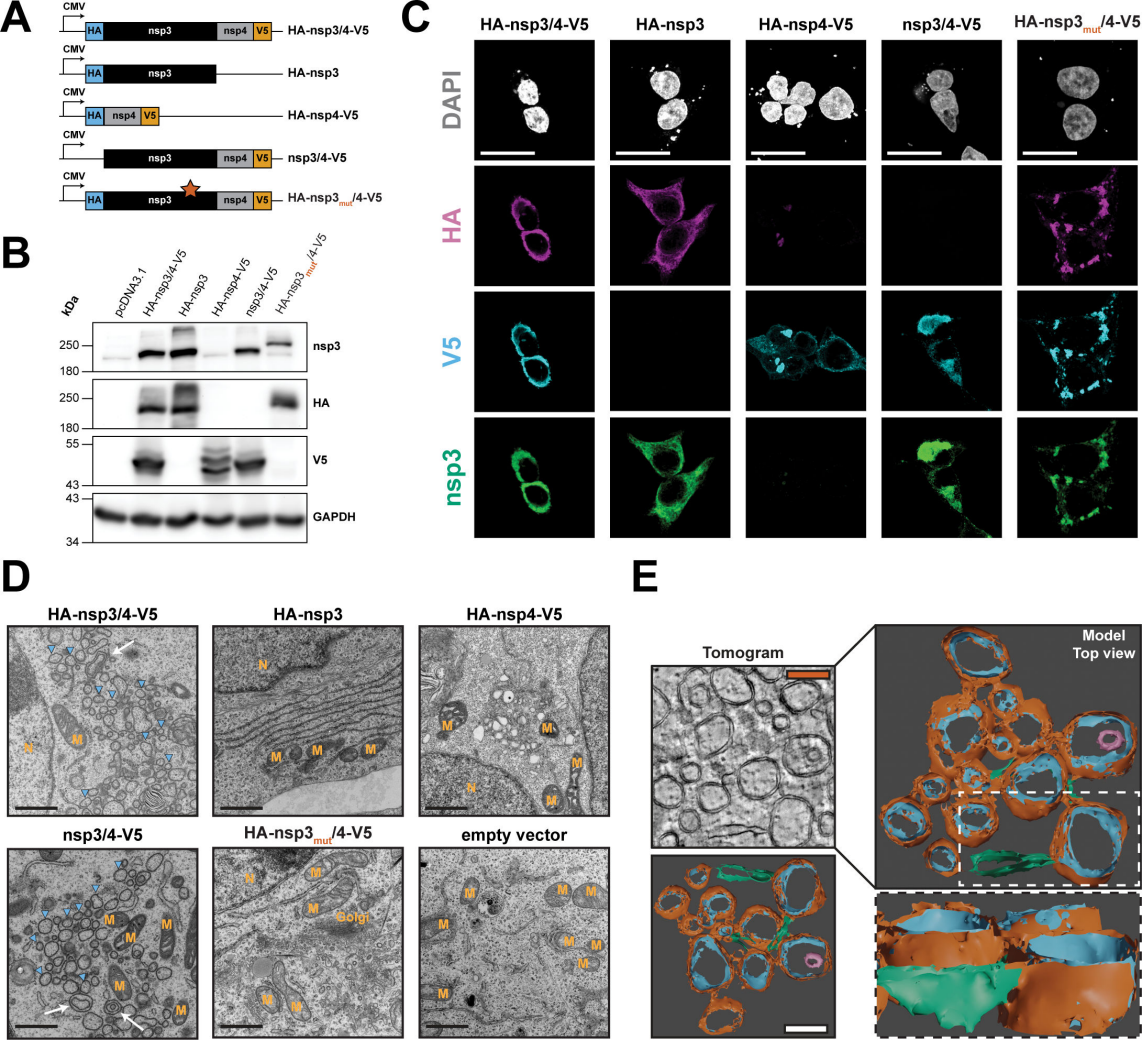
Characterization of SARS-CoV-2 nsp3 and nsp4 in context of single and polyprotein expression. (**A**) Schematic overview of the expression constructs used in this study. Expression of the viral proteins is driven by the cytomegalovirus (CMV) immediate early promoter. The HA-nsp3_mut_/4-V5 construct contains the alanine substitution C1592A (star) that abrogates PLpro protease activity. (**B**) HEK293T cells were transfected with the given expression constructs, and 24 hours later, cells were lysed, and HA-nsp3- and nsp4-V5-tagged proteins were detected by western blot. GAPDH served as loading control; protein molecular mass in kilodalton is given on the left. (**C**) HEK293T cells grown on glass cover slips were transfected with the indicated constructs and fixed 24 hours post transfection. Immunostaining was performed with specific antibodies raised against nsp3, the HA epitope or the V5 epitope. Subcellular localization was visualized by confocal microscopy using a Leica SP8 confocal microscope and lightning deconvolution. Scale bars = 20 µm. (**D**) HEK293T cells were transfected with the indicated constructs and fixed with electron microscopy fixative 24 hours post-transfection before being processed for transmission electron microscopy analysis using a Jeol JEM-1400 electron microscope. Images of representative fields of view are shown. Blue arrowheads indicate DMVs, and white arrows indicate multimembrane vesicles. M, mitochondria; N, nucleus; Golgi, Golgi apparatus. Scale bars = 1 µm. (**E**) HEK293T cells were transfected with the HA-nsp3/4-V5 construct. After 24 hours, samples were fixed, embedded in epoxy resin, and a tilt-series was acquired using a FEI Tecnai F20. After alignment, 3D surface models were generated by segmentation in Ilastik and rendered using blender software. Scale bars = 250 nm. Orange, DMV outer membrane; blue, DMV inner membrane; magenta, inner multimembrane vesicle; green, ER.

To determine the role of nsp3 and nsp4 in SARS-CoV-2-induced membrane remodeling independent of virus replication, we generated expression constructs with codon-optimized sequences (17, 32) (Fig. 1A). Hemagglutinin (HA) and V5 affinity tags were fused to the N-terminus of nsp3 (HA) and the C-terminus of nsp4 (V5), respectively. In addition to the nsp3/4 polyprotein constructs, nsp3 and nsp4 single-encoding constructs were generated for the investigation of individual protein function. Furthermore, a polyprotein construct lacking the N-terminal HA tag was generated as a technical control for downstream proteomic analysis. As a biological control, we genetically inactivated the protease active site located in nsp3, which mediates cleavage at the nsp3/4 junction and is reported to be required for membrane remodeling of related coronaviruses (15).

Protein expression in HEK293T cells following transfection with the indicated constructs was confirmed by western blot analysis (Fig. 1B). Both nsp3- and HA-specific antibodies demonstrated the expression of proteins with the expected molecular mass for nsp3 (~220 kDa), for HA-nsp3/4-V5 and HA-nsp3. V5-tagged nsp4 was also detected at the expected size (~55 kDa) indicating proper polyprotein processing. In lysates from cells expressing the protease mutant construct (HA-nsp3_mut_/4-V5), we observed a single band at a high molecular weight and no signal for cleaved nsp4-V5, indicating that inactivation of the protease activity resulted in the expression of an unprocessed polyprotein at a higher molecular weight compared to nsp3 alone due to the retention of nsp4. For the nsp3/4-V5 construct lacking the HA epitope, anti-nsp3 antibody staining revealed a band at the expected molecular mass, while no signal was present when the membrane was probed with the anti-HA antibody. Western blot analysis of lysates from cells expressing the HA-nsp3/4-V5 and nsp3/4-V5 constructs revealed bands at the expected molecular weight for nsp4-V5. In lysates from cells expressing the single-protein construct HA-nsp4-V5 with no expression of nsp3, two bands were detected with the V5-specific antibody. Such profiles have been previously described and were attributed to different nsp4 glycosylation states (15, 33, 34).

We next evaluated the subcellular localization of viral proteins in the transfected HEK293T cells (Fig. 1C). HA and V5 affinity tag-specific antibodies as well as nsp3-specific antibodies were used to probe viral protein localization by immunofluorescence analysis. In cells expressing HA-nsp3/4-V5, we observed perinuclear clusters of high-intensity fluorescence signals that overlapped for all three antibodies. A similar staining pattern was observed upon expression of the nsp3/4-V5 polyprotein construct when detected with the nsp3- or V5-specific antibodies, but no signal was detectable for HA-specific antibodies. When expressed individually, nsp3 and nsp4 showed primarily reticular staining patterns, although clusters of high-intensity signal were observed repeatedly in cells expressing nsp4-V5 alone. Only low-intensity signal could be detected for the HA-specific antibody staining in nsp4-transfected cells, which might be due to the membrane-proximal position of this tag. Expression of the polyprotein construct with the mutated protease active site in nsp3 resulted in the formation of high-signal-intensity puncta in the cytosol, indicating that protease-mediated cleavage of nsp3/4 is required for proper subcellular localization.

### Electron microscopy reveals extensive SARS-CoV-2 nsp3/4-mediated endomembrane remodeling that parallels virus infection

To further investigate membrane remodeling induced by SARS-CoV-2 nsp3/4, we used electron microscopy (EM) to resolve membrane structures in cells expressing the various nsp3/4 constructs (Fig. 1D). Expression of the nsp3/4 polyprotein constructs with intact protease induced extensive changes in the endomembrane system that paralleled the changes seen during virus infection. We observed clusters of DMVs of different sizes and, at a lower frequency, multimembrane vesicles (Fig. 1D, blue arrowheads and white arrows, respectively). The DMV clusters often resided in the perinuclear region and in proximity to mitochondria. Paralleling our fluorescence microscopy data, in cells expressing the nsp3/4 polyprotein with an inactive protease, we did not observe the formation of structures resembling DMVs. Expression of the single protein constructs did not show consistent changes in the endomembrane system, although vesicle aggregations could be observed in some nsp4-expressing cells.

To gain a better understanding of the three-dimensional architecture of nsp3/4-induced membrane alterations, we next generated a three-dimensional model of the nsp3/4-induced DMVs by acquiring a tilt-series of 250-nM-thick sections by electron tomography. The tomographic model revealed connections between the individual DMVs through paired membranes (Fig. 1E, orange and blue, respectively) and clusters of extensively remodeled membranes in all dimensions. This included connected DMVs stacked on top of each other and multimembrane vesicles (Fig. 1E, magenta). Additionally, we observed connections between ER membranes (green) and the outer membrane of the DMVs similar to what was previously described in virus infection (10). As reported earlier, the diameter of the DMVs in the absence of viral replication was smaller compared to virus-infected cells [160 nm versus 300 nm; (16)], which might be due to the absence of replicating viral RNA in the expression system used in this study. In summary, the sole expression of SARS-CoV-2 nsp3/4 is sufficient to induce the formation of double membrane and multimembrane vesicles with morphologies that parallel replication-competent DMVs observed in virus-infected cells, albeit with a reduction in size.

### Abundance and localization of organelle marker proteins are altered in SARS-CoV-2 nsp3/4-expressing cells

**Fig 2:**
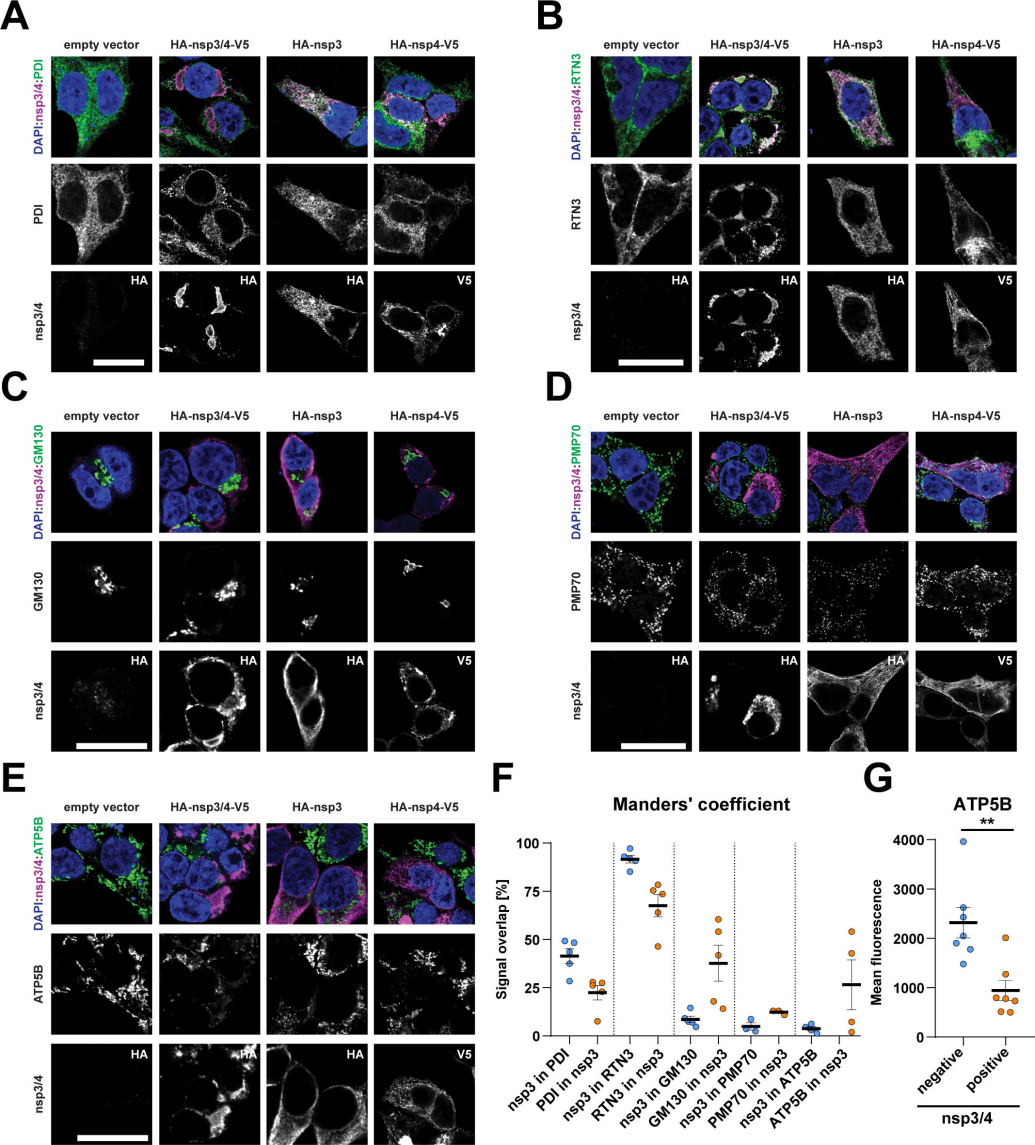
Abundance and localization of organelle marker proteins are altered in SARS-CoV-2 nsp3/4-expressing cells. (**A–E**) HEK293T cells were transfected with the indicated constructs and fixed 24 hours post transfection. Viral proteins were detected using specific antibodies against the HA or V5 epitope tags (nsp3/4) as specified in the lower image row of each panel. Endoplasmic reticulum markers were detected by staining of protein disulfide-isomerase and RTN3 (A and B, respectively). Golgi apparatus was detected by using GM130 (**C**), peroxisomes with PMP70 (**D**), and mitochondria by staining for ATP synthase subunit 5B (**E**). Scale bars = 20 µM. (**F**) Quantification of co-localization of HA-nsp3 and organelle marker signal shown as mean ± SEM. (**G**) Quantification of intracellular ATP5B signal intensity per cell in nsp3/4-negative (blue) and -positive cells (orange). An unpaired *t*-test was performed to assess statistical differences with *P < 0.05, **P < 0.01, ***P < 0.001.

Due to extensive alterations of the ER, we were next interested in which other compartments and structures in the cell might be altered in nsp3/4-expressing cells and if specific recruitment of host cell proteins can be observed. Previous studies showed that the ER-resident protein reticulon 3 (RTN3) is recruited to sites of virus replication, while another ER-localized enzyme, protein disulfide isomerase (PDI), was not recruited (10, 16, 26). Therefore, we performed immunofluorescence analysis of these ER markers in cells expressing nsp3/4, or individual nsp3 or nsp4 as controls, to evaluate alterations in ER morphology (Fig. 2A and B). We observed a strong overlap in fluorescence signal between viral proteins and PDI or RTN3 in cells expressing the individual viral proteins, consistent with minimal effect on ER remodeling, but no significant changes in subcellular localization of either cellular ER marker. Although expression of the nsp3/4 polyprotein construct did not change the localization of PDI (Fig. 2A), subcellular localization of RTN3 strikingly overlapped with nsp3/4 signal, arguing for recruitment to sites enriched for both nsp3 and nsp4 fluorescent signals (Fig. 2B). We further studied the subcellular localization of Golgi network, peroxisome, and mitochondrial markers but did not observe changes in localization of GM130 (Golgi network), PMP70 (peroxisomes), or the mitochondria-resident protein ATP synthase subunit 5B (ATP5B) (Fig. 2C through E). Co-localization was quantified and confirmed a high signal overlap between nsp3 and RTN3 in nsp3/4-expressing cells, while the remaining markers did not exhibit such an overlap (Fig. 2F). For ATP5B, despite the very low signal overlap with nsp3 (Fig. 2F), we detected a reduced mean fluorescence intensity in nsp3/4-expressing cells when using this single-cell-based readout (Fig. 2G). This observation is consistent with an earlier study of infected cells revealing structural and functional perturbation of mitochondria (10), and our data suggest that nsp3/4 contributes to this perturbation. In summary, these results show that nsp3 and nsp4 localize to the ER and that upon nps3/4 expression, a subset of ER-resident proteins is recruited to sites enriched for these viral proteins. Furthermore, while the localization of markers of the Golgi network and peroxisomes seemed unaltered, the abundance of the mitochondria-resident protein ATP5B was negatively affected by nsp3/4 expression.

### Interactome of SARS-CoV-2 nsp3 and nsp4 in context of single and polyprotein expression

**Fig 3:**
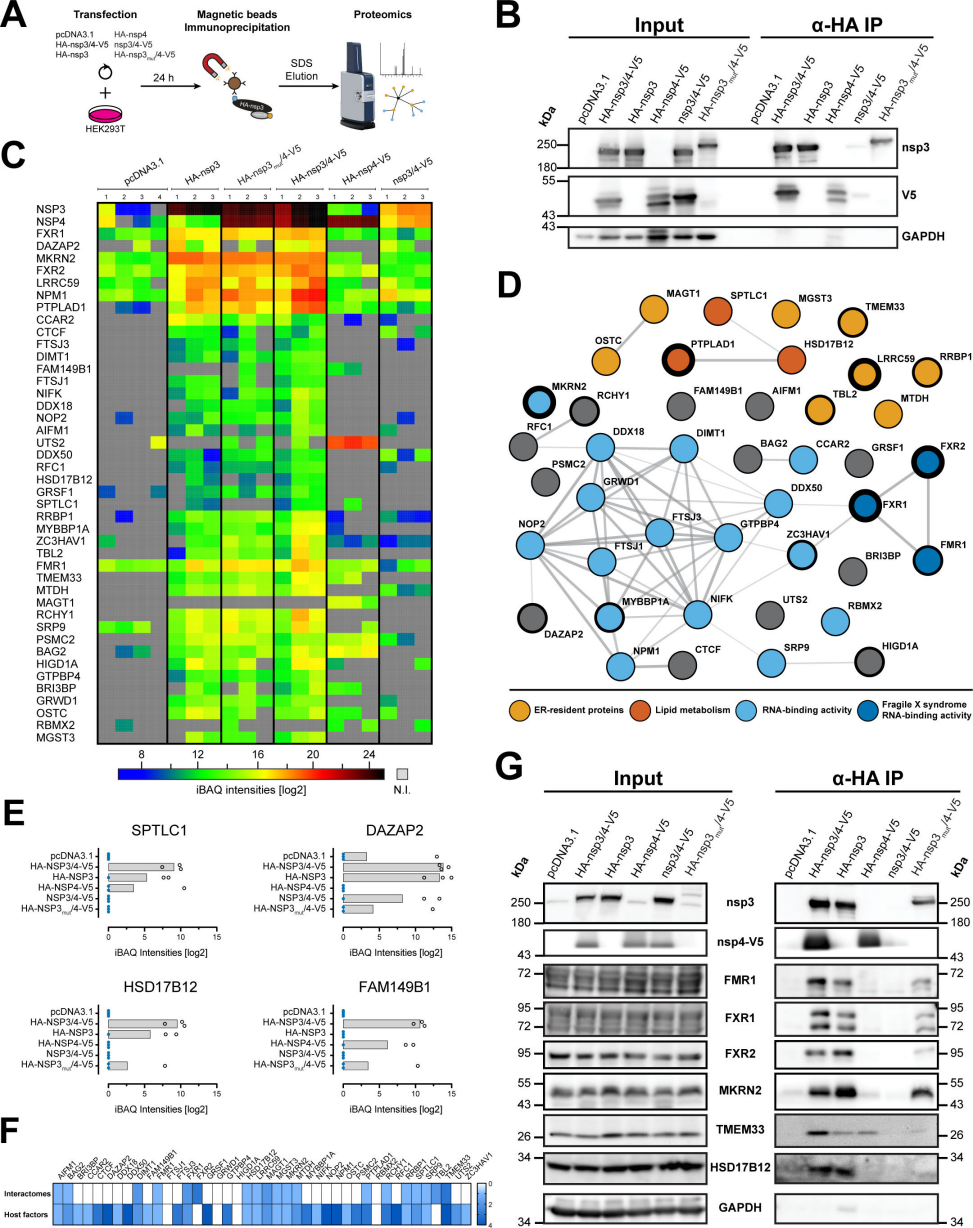
Identification of nsp3/4 interaction partners by mass spectrometry analysis of co-immunoprecipitated proteins. (**A**) Schematic overview of the immunoprecipitation experiments. HEK293T cells seeded into 10-cm-diameter dishes were transfected with the described constructs and 24 hours post-transfection, cells were lysed using buffer containing dodecylmaltoside that was necessary to allow efficient capture of viral proteins. Lysates were mixed with HA-specific magnetic beads for immunoprecipitation and HA-tagged proteins, and their interactors were eluted with 3% SDS in Tris-HCl. Eluates were analyzed by label-free liquid chromatography coupled to mass-spectrometry. (**B**) Nsp3- and V5-tagged proteins were detected by western blot. GAPDH served as a loading control. IP experiments were performed in biological quadruplicates, and exemplary western blots are shown. (**C**) Heatmap of selected proteins identified in AP-LC-MS/MS analysis. Intensity-based absolute quantification (iBAQ) intensities are indicated by color according to the scale shown on the bottom (N.I. = not identified). (**D**) String network analysis of the identified interaction partners. Proteins resident at the ER (light orange), involved in lipid metabolism (dark orange), having RNA-binding activity (light blue), and proteins of the Fragile X syndrome family (dark blue) were identified by gene ontology analysis and are highlighted by colored circles specified on the bottom. The thickness of the circle boundaries indicates the level of enrichment in the HA-nsp3/4-V5 sample set. (**E**) Bar graphs of selected proteins specifically enriched in the wild-type HA-nsp3/4-V5 pull-downs compared to the inactive protease mutant control. Missing values are indicated by blue dots. (**F**) Comparison of the interaction partners determined in this study with interaction partners described in other proteomic data sets of SARS-CoV-2 nsp3 and nsp4 (top row) and with statistically significant host cell factor hits reported in genome-wide screens for SARS-CoV-2 (bottom row). The number of data sets reporting a given interaction or dependency factor is shown as a heatmap indicated on the right. (**G**) Interaction of the cellular proteins identified by mass spectrometry was validated by pull-down and western blot analysis of captured protein complexes using western blot and antibodies of given specificity. Immunoprecipitation was confirmed by nsp3 and nsp4-V5 detection. GAPDH served as a control of specific enrichment of immunoprecipitated proteins. Protein molecular weights (in kilodalton) are given on the left and right sides of the blots.

To identify host proteins potentially involved in SARS-CoV-2 nsp3/4-mediated membrane reorganization, we determined cellular interaction partners using affinity purification liquid chromatography coupled to mass-spectrometry (AP-LC-MS/MS). To streamline identification of host proteins potentially involved in DMV biogenesis or function, we included the individual cleavage products of the polyprotein (nsp3 and nsp4) as well as the PLpro catalytically inactive nsp3/4 polyprotein in our analysis (Fig. 3A). Immunoprecipitation (IP) efficiency was first evaluated by western blot, and specificity and sensitivity were confirmed for each of the individual experiments and samples subsequently processed for LC-MS/MS (Fig. 3B).

Mass spectrometry analysis revealed more than 200 host proteins significantly interacting with nsp3, nsp4, and/or nsp3/4 when compared to the empty vector control (Student’s *t*-test; *P* < 0.05, false discovery rate = 0.05; Table S1). Among these, we identified a distinctive enrichment of ribosomal proteins, as well as proteins involved in lipid metabolism and members of the transmembrane protein family (TMEM), consistent with a cellular microenvironment supportive of ROs. For functional downstream studies, we focused on host proteins selectively enriched in nsp3/4 when compared to the untagged counterpart or nsp4 alone, deliberately excluding ribosomal proteins (*n* = 44, Fig. 3C). String network analysis of all selected hits combined with gene ontology analysis allowed us to determine clusters of proteins with similar function and/or previously described interactions (Fig. 3D). This revealed a high degree of interconnection between the selected factors and showed enrichment for ER-resident proteins, factors involved in lipid metabolism, and RNA-binding proteins. Interestingly, three members of the protein family associated with the Fragile X syndrome, which possess RNA binding activity, were identified. Other gene ontology terms enriched in our list of interaction partners included RNA methyltransferase activity, biosynthesis of unsaturated fatty acids, regulation of cellular catabolic processes, and response to ER stress. Furthermore, four factors were robustly and selectively enriched in wild-type HA-nsp3/4-V5 pull-downs when compared to the inactive protease counterpart (Fig. 3E).

For further validation, we performed an analysis of previously published interaction studies (34 – 37) and reports describing host dependency factors identified via genome-wide screening (28, 38 – 40). Comparison with our data set revealed some overlapping interaction partners, but also demonstrated that our approach identified new host proteins (Fig. 3F). Interestingly, the majority of cellular interaction partners newly identified in this study were reported to affect virus replication in the published CRISPR screens.

Prioritized host-interacting proteins identified by mass spectrometry were further validated by immunoprecipitation and western blot analysis using host cell factor-specific antibodies readily available to us (Fig. 3F). In this way, we confirmed the interaction of FMR1, FXR1, and FXR2 of the Fragile X syndrome family as well as the E3 ubiquitin ligase MKRN2 with nsp3, both in context of single and nsp3/4 expression. Furthermore, interaction of the transmembrane protein TMEM33 with nsp3 and nsp4 was validated in both individual protein and polyprotein-expressing cells. In agreement with the mass spectrometry analysis, we confirmed that HSD17B12, an enzyme involved in fatty acid metabolism, specifically interacts with nsp3 only when the protease was active. Taken together, our data demonstrate that several host cell factors interact with SARS-CoV-2 nsp3 and nsp4 in the context of single and polyprotein expression, pointing toward specific host cell proteins and pathways that might be involved in RO formation or function.

### Functional interrogation of SARS-CoV-2 nsp3/4 interaction partners

**Fig 4:**
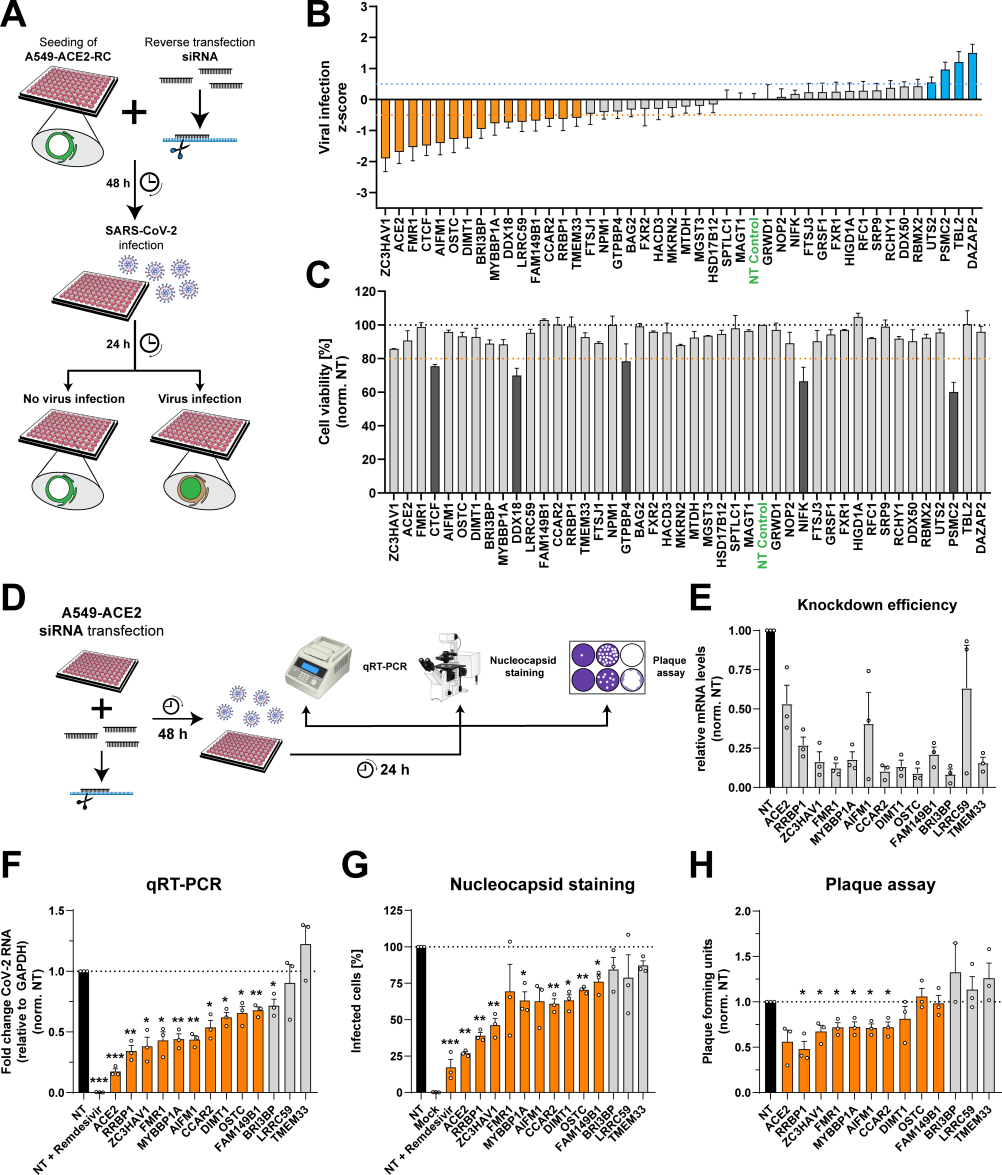
Functional role of SARS-CoV-2 nsp3/4 interaction partners in the viral replication cycle. (**A**) Schematic overview of the siRNA-mediated gene silencing approach used for the investigation of the role of SARS-CoV-2 nsp3/4 interaction partners in the viral replication cycle. A549 cells constitutively expressing the virus receptor ACE2 and a reporter construct (A549-ACE2-RC) were seeded into glass-bottom 96-well plates for microscopy analysis and reverse transfected with siRNA pools targeting the identified interaction partners. And 48 hours post-transfection, cells were infected with SARS-CoV-2 (MOI = 5) and, after 24 hours, fixed using formaldehyde. The percentage of infected cells upon knockdown of a given interaction partner was evaluated with a semi-automated image analysis pipeline. (**B**) Summary of two independent experiments, each performed with technical triplicates. The *z*-score calculated for the different interaction partners and the controls targeting the virus receptor ACE2 and a non-targeting control siRNA (green) are shown as a mean ± SEM. Cutoffs were set at a mean *z*-score of ±0.5 (dotted lines), and host dependency and restriction factors are colored in orange and blue, respectively. (**C**) In parallel to the experiments described in (**B**), A549-ACE2-RC cells were transfected with siRNAs, the media were exchanged 48 hours post-transfection, and the cell viability was determined at 72 hours post transfection using the CellTiter Glo assay. The reduction of cell viability by gene silencing was considered cytotoxic at values lower than 80% (orange-dotted line). (**D**) Schematic overview of the host factor validation assays. A549-ACE2 cells were seeded into 24-well plates and transfected with siRNA pools targeting host factors identified by the reporter cell screen. Forty-eight hours later, cells were infected with SARS-CoV-2 (MOI = 5). Also, 24 hours post infection, supernatant was collected for plaque-forming assay, and cells were either fixed in 10% formaldehyde for immunofluorescence assay, or RNA was extracted for qRT-PCR to determine knockdown efficiency (**E**) or viral RNA levels (**F**). (**G**) Quantification of infected cells was performed by immunofluorescence staining of the viral nucleocapsid protein. The percentage of infected cells was normalized to the non-targeting control and plotted. (**H**) Release of infectious virus particles was measured by plaque-forming assay with serially diluted supernatants harvested 24 hours post infection. Data were normalized to the non-targeting control. Orange bars indicate significant reduction of viral replication in more than two assays. The experiments were performed in biological triplicates, and data were plotted as mean ± SEM. An unpaired *t*-test was performed to assess statistical differences with *P < 0.05, **P < 0.01, ***P < 0.001.

We next investigated the functional requirement for each of the interaction partners in the SARS-CoV-2 infection cycle and performed a small-interfering RNA (siRNA)-based gene silencing approach. To this end, we employed a previously described reporter cell line that utilizes nuclear translocation of a fluorescent protein to identify virus-infected cells (Fig. 4A) (41). Forty-eight hours after reverse transfection of siRNA pools targeting RNAs of the identified interaction partners, cells were infected with SARS-CoV-2 for 24 hours prior to fixation and imaging. The *z*-score for each target was calculated using a semi-automated image analysis pipeline to identify the percentage of infected cells (Fig. 4B). In parallel, cell viability upon gene silencing was assessed using CellTiterGlo assay quantifying cellular ATP content as an approximation for cell metabolism (Fig. 4C). Importantly, our non-targeting control siRNA pool showed a *z*-score close to 0, while our positive control, an siRNA pool targeting the virus receptor ACE2, showed a *z*-score of −1.7 (± 0.9). Based on this range, we considered targets as potential host restriction and dependency factors at a *z*-score threshold of ±0.5 as indicated in blue and orange in Fig. 4B, respectively. After exclusion of hits that decreased cell viability (Fig. 4C, dark gray) and the positive control ACE2, this analysis led to the identification of 12 host dependency factors and 3 possible host restriction factors for SARS-CoV-2.

To determine host factors contributing to DMV biogenesis, we focused on the dependency factors and validated their function in the full viral life cycle in orthogonal experimental approaches (Fig. 4D). Paralleling reporter cell line experiments described above, we transfected A549-ACE2 cells with siRNA pools targeting the genes of interest and infected the cells 48 hours later with SARS-CoV-2. Twenty-four hours post infection, total cellular RNA was collected for siRNA silencing validation (Fig. 4E) and quantification of intracellular viral RNA (Fig. 4F). In parallel, cells were fixed and stained for viral nucleocapsid protein to determine the percentage of infected cells (Fig. 4G), while supernatants were collected to assess release of infectious viral particles by plaque assay (Fig. 4H). We observed a significant reduction in intracellular viral RNA levels, percentage of infected cells, and release of infectious viral particles for our ACE2 siRNA control and for cells treated with the antiviral nucleoside analog Remdesivir. We could not confirm reduced viral replication for three of the previously identified factors (BRI3BP, LRRC59, and TMEM33) with the caveat that LRRC59 knockdown was insufficient (Fig. 4E). Importantly, depletion of the other identified host cell factors led to a reduction of virus replication or release of viral particles, although less prominent in the latter assay (Fig. 4F through H, orange bars). While these results validated our reporter cell line approach, obtained data indicate that several of the identified SARS-CoV-2 nsp3/4 interactors play an important role for virus replication and spread.

### Ultrastructural analysis reveals the importance of FAM149B1, CCAR2, and ZC3HAV1 for DMV formation

Next, we determined whether the identified nsp3/4 interactors are involved in viral RO formation. HEK293T cells were transfected with siRNA pools targeting the respective genes and 2 days later transfected with the HA-nsp3/4-V5 construct. We used a non-targeting siRNA pool as a negative control in which the knockdown procedure was not expected to affect DMV formation.

**Fig 5:**
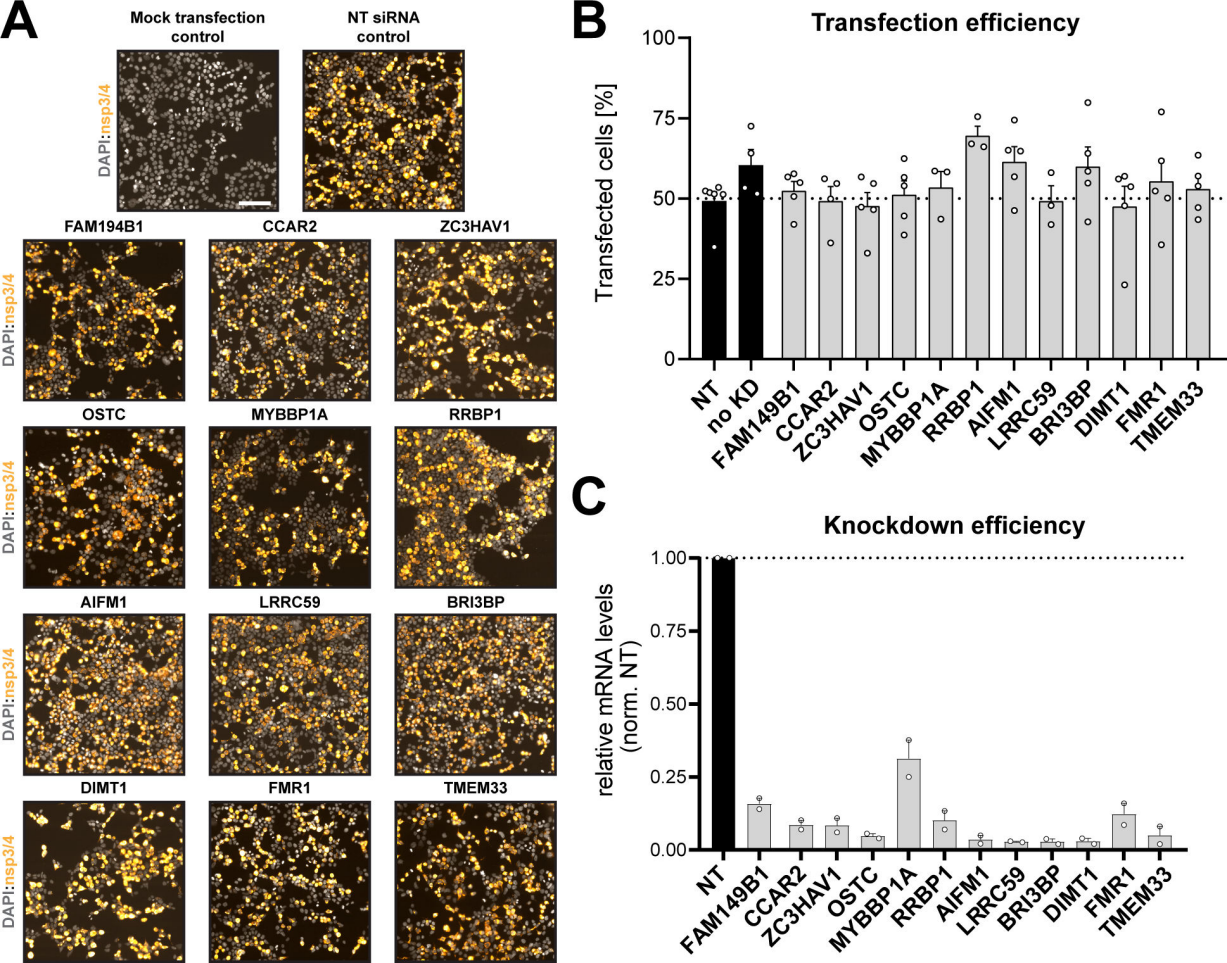
Validation of transfection efficiency and impact of knockdown on nps3/4 expression. (**A and B**) HEK293T cells seeded onto coverslips in a 24-well plate were transfected with siRNAs targeting the given host dependency factors. And 48 hours post-transfection, the medium was replaced, and cells were transfected with the HA-nsp3/4-V5 expression construct. Cells were fixed 18 hours later using paraformaldehyde (4%) and processed for IF analysis. Transfection efficiency was determined by staining with HA-specific antibodies and counting of positive cells. Representative fields of view are displayed. Scale bar = 20 µM. (**C**) HEK293T cells were reverse transfected with siRNA pools and treated as described above. And 72 hours post transfection, cells were lysed, RNA was isolated, and given mRNAs were quantified by real-time PCR. GAPDH served as housekeeping gene and was used to normalize values. Data shown as mean ± SEM normalized to fold over the NT control from three independent experiments.

**Fig 6:**
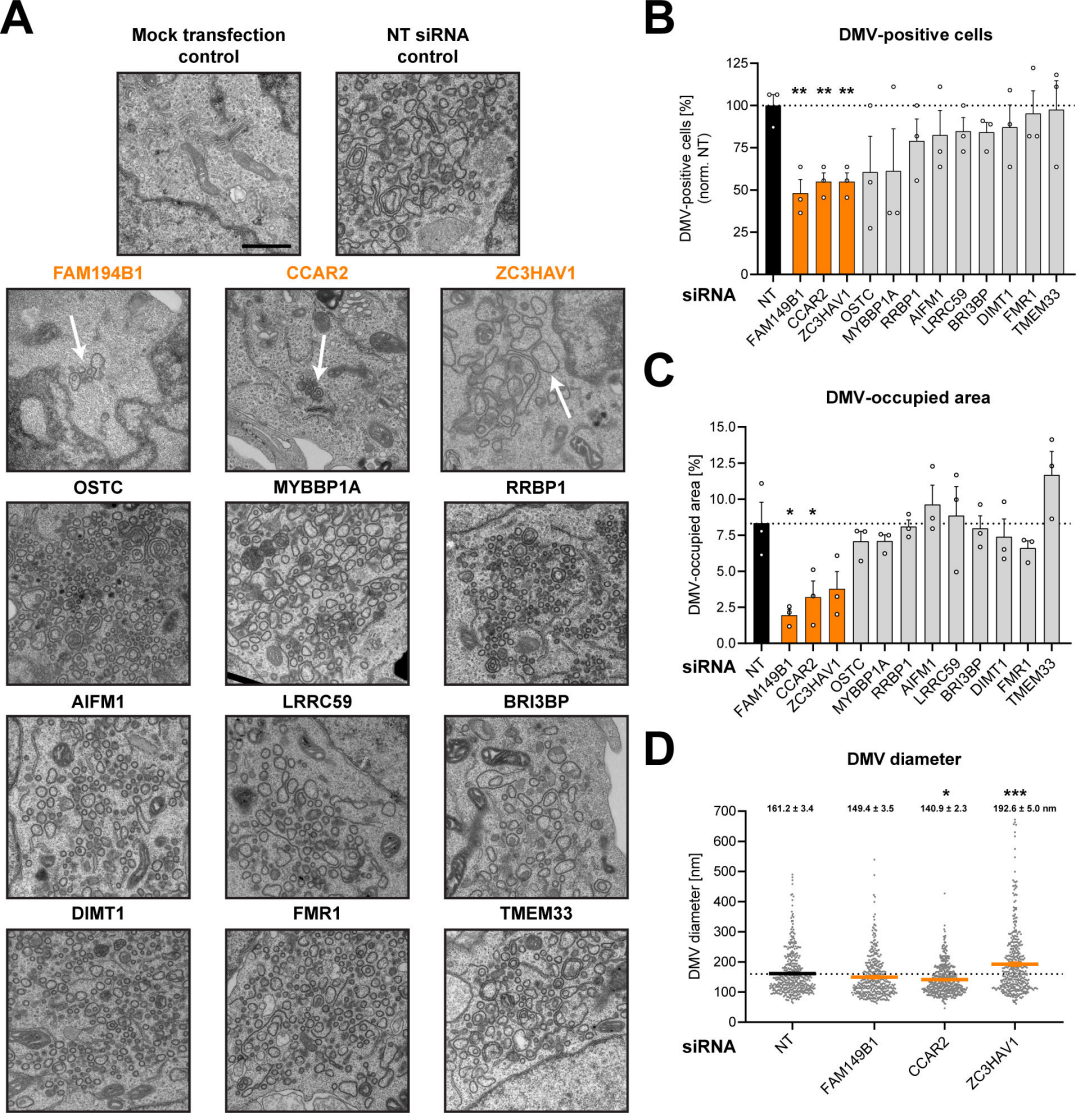
FAM149B1, CCAR2, and ZC3HAV1 are host dependency factors involved in the biogenesis of SARS-CoV-2 nsp3/4-induced replication organelles. (**A–D**) HEK293T cells seeded onto coverslips in a 24-well plate were transfected with siRNA targeting the respective host dependency factors. And 48 hours post-transfection, the medium was replaced, and cells were transfected with the HA-nsp3/4-V5 expression construct. Also, 24 hours later, cells were fixed using EM fixative and processed for TEM. Overview images of the grid hexagons were taken at 200× magnification using SerialEM. Two micrographs per cell were acquired from all cells within the hexagon at a 10.000× magnification to assess DMV formation. (**A**) Representative images of DMVs are shown with white arrows indicating the few DMVs present in cells treated with siRNA targeting FAM149B1, CCAR2, and ZC3HAV1. DMV-positive cells (**B**) and the area occupied by DMVs (**C**) were quantified for at least 20 cells per replicate, and values were normalized to the NT control. (**D**) The diameter of DMVs present in cells treated with the NT siRNA control or with siRNAs targeting FAM149B1, CCAR2, and ZC3HAV1 was quantified for at least 75 DMVs per replicate. The experiments were performed in biological triplicates, and data are plotted as mean ± SEM. An unpaired *t*-test was performed to assess statistical differences with *P < 0.05, **P < 0.01, ***P < 0.001.

We first assessed the transfection efficiency of our HA-nsp3/4-V5 expression construct (Fig. 5A and B) and found that ~50%–70% of the cells were transfected with each construct. This efficiency was sufficient for the planned analysis by transmission electron microscopy (TEM) for which an efficiency of approximately 50% was required to ascertain that detection of DMVs was not flawed by too low number of nsp3/4-expressing cells. Moreover, we confirmed that siRNA knockdowns did not affect expression of viral proteins (Fig. 5A and B) and evaluated knockdown efficiency (Fig. 5C). In parallel to the immunofluorescence (IF) analysis, the cells were processed for TEM to assess the formation of DMVs after the depletion of the target protein expression (Fig. 6A through D). In the control samples transfected with the non-targeting siRNA pools, we readily detected extensive ER remodeling and the formation of DMVs, while no such changes were found with the untransfected control cells (Fig. 6A). We then randomly selected at least 20 cells per replicate (based on transfection efficiency corresponding to around 10 cells per replicate expressing nsp3-4) and quantified the percentage of DMV-positive cells (Fig. 6B) and the area occupied by DMVs in all imaged cells per sample (Fig. 6C). We found that knockdown of most factors did not alter DMV formation as deduced from the percentage of DMV-positive cells (Fig. 6B) and DMV-occupied area in these cells relative to the control cells. Strikingly, clusters of DMVs were significantly less frequent and occupied a smaller area in cells transfected with siRNA pools targeting FAM149B1, CCAR2, and ZC3HAV1 (Fig. 6C). To gain insight into potential morphological alterations of these few formed DMVs, we quantified the diameter of at least 75 DMVs per replicate (Fig. 6D). While we did not observe a significant difference in DMV size in cells depleted for FAM149B1, CCAR2 depletion led to a reduced diameter of the detected DMVs, while knockdown of ZC3HAV1 resulted in the formation of larger, more spherical DMVs (Fig. 6A, white arrow). These data suggest that the nsp3/4 interaction partners FAM149B1, CCAR2, and ZC3HAV1 are SARS-CoV-2 host dependency factors that appear to play a role in remodeling of the endomembrane system to form viral replication organelles.

## DISCUSSION

In this study, we utilized a transfection-based system to induce SARS-CoV-2 ROs independent of viral replication. Confocal light microscopy as well as TEM and electron tomography confirmed the generation of DMVs that are morphologically similar to those observed in SARS-CoV-2-infected cells (10, 11, 16). In addition, our host protein interactome data set of full-length nsp3 and nsp4 in single and polyprotein context provides further insight into SARS-CoV-2 host factors compared to available proteome data. Furthermore, we report interaction partners in the presence and absence of nsp4 and nsp3 protease activity, adding additional layers of information and expanding the view on SARS-CoV-2 host factor interactions.

Characterization of the SARS-CoV-2 nsp3/4 expression system confirmed proper production of the viral proteins (Fig. 1B). The two bands observed when nsp4 was expressed individually were also previously reported and have been suggested as different glycosylation states (33, 34). Interestingly, the second band was not found in context of nsp3/4 polyprotein expression indicating that nsp3 might have an impact on the glycosylation and/or folding status of nsp4. Immunofluorescence imaging demonstrated that nsp3 and nsp4 expressed on their own exhibited a mostly reticular staining pattern consistent with their ER localization, while the nsp3/4 polyproteins were found in perinuclear, high signal intensity clusters (Fig. 1C). Importantly, the inactivation of the protease active site located in the PLpro domain of nsp3 altered subcellular localization and led to the formation of smaller clusters reminiscent of inclusion bodies.

Ultrastructural analysis by TEM revealed extensive alterations of the ER following expression of the HA-nsp3/4-V5 polyprotein (Fig. 1D). We observed highly interconnected clusters of DMVs, with DMV diameter being smaller in size compared to DMVs observed in infected cells, which might be due to the absence of viral full-length and sub-genomic RNAs or other viral proteins (11, 13, 16). Electron tomography and three-dimensional modeling allowed for the visualization of the highly clustered reticulovesicular network with connections between DMVs in all three dimensions (Fig. 1E). Additionally, continuity of the outer membrane of the DMVs with the ER by narrow connector structures was evident. HA-nsp3/4-V5-induced DMVs, despite their smaller size, exhibited an overall similarity to the ones observed in MHV and SARS-CoV-2-infected cells (10, 11). In addition to DMVs, multimembrane vesicles could also be observed, although at a lower frequency. Structures like DMSs and CMs that were reported in infected cells were not observed upon HA-nsp3/4-V5 expression in HEK293T cells or too infrequent to be detected in the expression system. These differences in membrane alterations between expression-based and infection systems are likely due to the presence of viral RNA or additional viral proteins such as nsp6 or the replicase complex in the infection setting. Our data and previous reports, therefore, suggest that while RNA and nsp6 can alter the lipid composition and size of the DMVs, they are not strictly required for DMV biogenesis that seems to be driven by nsp3/4 (19, 42). Additionally, cell line-specific differences may also contribute to variation in membrane alterations as very few DMSs were observed in SARS-CoV-2-infected lung cells (10). Importantly, DMSs and CMs have been reported to not contain dsRNA or newly synthesized RNA and are therefore likely not involved in virus replication (12).

RTN3 was recruited to sites positive for nsp3/4, while the localization of PDI remained unaltered (Fig. 2A and B). Recruitment of RTN3 to sites of viral replication was previously reported for SARS-CoV-2-infected cells, although the role of nsp3/4 so far was unclear (10). Reticulons have been proposed as important host factors due to their membrane-shaping activity (43), and a recent study implicated them in replication organelle formation (26).

Immunoprecipitation of HA-tagged viral proteins was combined with mass spectrometry to identify a set of 42 cellular interaction partners in single-protein and polyprotein context (Fig. 3C). Comparison of the list of selected interaction partners with previously reported host factors revealed that 40% of our hits were reported as host factors involved in SARS-CoV-2 infection in different data sets (28, 38). The majority of identified host interaction partners associate specifically with nsp3, while a smaller subset is associated with nsp4. Further analysis revealed four proteins that were only associated with nsp3 when the protease domain was active to separate proteolytically nsp3 from nsp4 (Fig. 3D). String network analysis allowed visualization of a cluster of related proteins involved in the Fragile X mental retardation (FMR) syndrome to specifically interact with nsp3/4. FMR protein 1 (FMR1) and fragile X mental retardation syndrome-related proteins 1 and 2 (FXR1/2) are RNA-binding proteins that regulate translation of the bound mRNAs (44). Members of this protein family were previously shown to interact with the hypervariable domain of the non-structural protein 3 of the alphavirus Venezuelan equine encephalitis virus (45). Interestingly, all CoV nsp3 proteins also contain a hypervariable region (8), suggesting that this domain might also be involved in FXR/FMR binding. Consistently, recent publications report that the FMR/FXR proteins interact with the N-terminal part of SARS-CoV-2 nsp3 (36) and directly with viral RNA (46).

In addition to the FMR family proteins, many other RNA-binding proteins were identified in our interactome analysis (Fig. 3D). This included Zinc finger CCCH-type antiviral protein 1 (ZC3HAV1) also known as zinc finger antiviral protein (ZAP), which has recently been shown to interact with SARS-CoV-2 RNA (46, 47). ZAP is expressed in several isoforms, which have been previously shown to act as restriction factor for a diverse set of viruses, including alphaviruses and filoviruses (48). While overexpression of the small isoform led to a slight reduction of viral RNA levels, overexpression of the long isoform did not affect replication of these viruses, although it was previously shown to be the more potent inhibitor of viruses (47, 49). In-depth analysis of our interactome data set revealed that the long isoform of ZC3HAV1 was the major interaction partner of nsp3, suggesting that the isoforms could have differential roles in SARS-CoV-2 infection. A possible hypothesis for the pro-viral role of ZC3HAV1 is that FXR1 has been reported as an interaction partner in previous studies (50, 51), indicating that the ZC3HAV1 and the FXR/FMR family members might be part of a multiprotein complex that interacts with nsp3/4. Additional work will be necessary to delineate the roles of the different isoforms and the role of the potential interaction of ZC3HAV1 with the FXR/FMR family in SARS-CoV-2 infection and DMV formation.

Besides ZC3HAV1, we identified the cell cycle and apoptosis regulator protein 2 (CCAR2) as an interactor of SARS-CoV-2 nsp3/4 that is potentially contributing to DMV formation. CCAR2 has been described as a transcriptional regulator involved in cell cycle control through its RNA-binding activity and interaction with deacetylases (52, 53). Interestingly, we observed interaction with nps3/4 in the absence of authentic viral RNA, and CCAR2 was not reported as a direct interactor of SARS-CoV-2 viral RNA (46, 47), indicating that the role of CCAR2 in the viral life cycle is not directly linked to its described RNA-binding activity. Interestingly, CCAR2 has also been shown to act as a major regulator of autophagy induction through inhibition of Sirtuin 1 (SIRT1) (54). A hypothesis on how CCAR2 might contribute to DMV formation might, therefore, be through its role in modulation of autophagy since components of the autophagy machinery have been reported to be involved in DMV formation (17). A potential link in this direction would be through the regulation of PI3K signaling, which is known to play a role in SARS-CoV-2 replication (17), as CCAR2 has been previously implicated in the regulation of the Akt pathway (55).

FAM149B1, the third nsp3/4 interactor contributing to DMV formation, is a poorly characterized protein and has not been previously implicated in SARS-CoV-2 infection. FAM149B1 is expressed in most cell types of the human body, and it interacts with numerous other cellular proteins involved in transport of amino acids, protein folding, or methylation of RNA. A previous report implicated FAM149B1 in the trafficking of specific proteins during cilium assembly (56). This regulatory role in protein trafficking might also be relevant for the transport of factors needed for DMV formation. Further studies will be required to determine how FAM149B1 contributes to SARS-CoV-2-induced DMV formation.

In summary, this study utilizes a plasmid-launched SARS-CoV-2 nsp3/4 expression system inducing RO formation allowing to characterize replication-independent mechanisms of DMV formation. We identify and validate host factors interacting with nsp3 and nsp4 and provide evidence for the importance of these factors in virus replication, revealing a potential role of FAM149B1, CCAR2, and ZC3HAV1 in the biogenesis of SARS-CoV-2 DMVs, the presumed sites of viral RNA synthesis.

This study has limitations. These include that knock-down of the identified host factors did not fully abrogate the formation of DMVs and virus replication. While this could be a result of an incomplete gene-silencing efficiency, validation of the functional relevance of these host cell factors will be needed in future studies through orthogonal assays, such as gene knockouts. Furthermore, while EM-based analysis is the most accurate approach to determine presence or absence of DMVs, the low throughput limits the number of cells analyzed and can therefore be skewed by technical and biological variations. Finally, a mechanistic link between the identified host factors and the biogenesis of DMVs is missing and will be topic of future studies.

## MATERIALS AND METHODS

### Cell lines and viruses

Human kidney HEK293T cells, African green monkey kidney VeroE6 cells, and human lung adenocarcinoma A549 cells were acquired from ATCC. A549-ACE2-RC cells constitutively expressing ACE2 and a reporter construct have been previously described (41, 57). Cells were cultured in Dulbecco’s modified Eagle medium (DMEM, Life Technologies) containing 10% fetal bovine serum (Sigma Aldrich), 100 U/mL penicillin, 100 µg/mL streptomycin, and 1% non-essential amino acids at 37°C and 5% CO_2_.

The SARS-CoV-2 isolate BavPat1 was kindly provided by Christian Drosten (Charité, Berlin, Germany) through the European Virology Archive. Virus stocks were generated by infection of VeroE6 cells and collection of supernatants. Virus concentration was determined by plaque assay.

### Virus titration

VeroE6 cells were infected with serial dilutions of supernatants harvested from infected cells. From 1.5 to 2 hours post infection, the medium was replaced by plaque medium containing 1.5% carboxymethylcellulose in MEM (Life Technologies). And 3 days post infection, cells were fixed with formaldehyde (5%), the cell monolayers were incubated with crystal violet (1%) in methanol (10%) for staining, and after extensive washing, plaques were counted. Infectious virus titers were calculated taking dilutions into account.

### DNA constructs

For the transfection-based polyprotein expression system, a codon-optimized sequence encoding SARS-CoV-2 non-structural proteins nsp3 and 4 was synthesized (BioCat) and transferred into a pcDNA3.1 expression vector. Coding sequences of affinity tags were added to the N- and C-termini of each viral protein allowing detection by antibodies. Nsp3 was tagged at the N-terminus with a hemagglutinin tag, while nsp4 was tagged with V5 at the C-terminus. Besides the full-length construct, the additional single-protein and polyprotein constructs were generated by PCR with the primers listed in Table 1. The active site of the nsp3 protease domain was inactivated by alanine substitution (C1592A) using the primers given in the primer list for overlap PCR.

**TABLE 1:** Primers

No.	Name	Sequence (5′ – 3′)
1	TMEM33_fwd	ACGGCAATGTGGCTTTCTC
2	TMEM33_rvs	CAGAGCACTGGTAAGAGCATTT
3	FMR1_fwd	ACTTACGGCAAATGTGTGCCA
4	FMR1_rvs	GCAGACTCCGAAAGTGCATGT
5	BRI3BP_fwd	CCGCTTCTTCTGGATCGTG
6	BRI3BP_rvs	GGACTGCTTCGCCAGTAGA
7	CTCF_fwd	CAGTGGAGAATTGGTTCGGCA
8	CTCF_rvs	CTGGCGTAATCGCACATGGA
9	OSTC_fwd	GTCCCGTTCTTAGTGCTCGAA
10	OSTC_rvs	CGACACTTGGAGGTTCAACAA
11	DDX18_fwd	GGAAGGCAGGGATCTTCTAGC
12	DDX18_rvs	CCACCCATTATCAAGCCATAGGT
13	DIMT1_fwd	GCTGGAGGACTCATGTTCAAC
14	DIMT1_rvs	CCTTGGGTCAAGTTCACAAGC
15	FAM149B1_fwd	ATCTACTGAAGGAAGCTCGGAC
16	FAM149B1_rvs	CACACTCAACTTCTGCTCATACA
17	AIFM1_fwd	GGCTTCCTTGGTAGCGAACTGG
18	AIFM1_rvs	GTCCAGTTGCTGAGGTATTCGG
19	CCAR2_fwd	CCTAGAACCTGCTGTCATCGCA
20	CCAR2_rvs	CTGGAGCATCTCCAGAAACAGC
21	LRRC59_fwd	TGACTACTCTACCGTCGGATTT
22	LRRC59_rvs	TTCAGGTCCAACCACTTCAGG
23	ZC3HAV1_fwd	CCGGTGCAACTATTCGCAGT
24	ZC3HAV1_rvs	TCAGTCCAGAGAGTTCGTGATTT
25	MYBBP1A_fwd	GACCGCTATGGCCTATTGAAG
26	MYBBP1A_rvs	GGGCATATTTCATCTCGGACC
27	RRBP1_fwd	TACGACACTCAAACCTTGGGG
28	RRBP1_rvs	GGTTGGCTAGGGCTTCTTCATA
29	DAZAP2_fwd	GATGCTCCACCTGCCTACTC
30	DAZAP2_rev	TGGAACCTAAAGGCCCAACA
31	GAPDH_fwd	GAAGGTGAAGGTCGGAGTC
32	GAPDH_rev	GAAGATGGTGATGGGATTTC
33	CoV-2 leader	TCCCAGGTAACAAACCAACCAACT
34	CoV-2 ORF7_rev	AAATGGTGAATTGCCCTCGT
35	nsp3 + HA_EcoRI_fwd	CCACTAGTCCAGTGTGGTGGAATTC
36	InactiveProt_P3_fwd	CGACAACAATGCCTACCTGGC
37	nsp4Ct_HAStart_fwd	AAAGAATTCATGGGCTACCCCTACGACGTGCCCGACTACGCCGGCACATGCGCCACCACCAG
38	ctNsp4_fwd	AAAGAATTCATGACATGCGCCACCACCAGG
39	CtNsp4_Stop_XbaI_rvs	AAATCTAGATCATTAGCCGGCGTAGTCGGGCACGTCGTAGGGGTAGCCAGGCAGCACGGCGC
41	nsp3_XbaI_rvs	AAAGTCTAGAGCCGCCCTTCAGGGC
42	nsp4_EcoRI_fwd	AAAGAATTCTACCCCTACGACGTGCCCGACTAAAGATCGTGAATAACTGGCTGAAGC
43	nsp4_XbaI_rvs	GGGTTTAAACGGGCCCTCTAGA
44	nsp3NoHASTART_EcoRI_fwd	AAAGAATTCATGGCTCCTACCAAGGTGACCTTCG
45	nsp4HASTART_EcoRI_fwd	AAAGAATTCATGGGCTACCCCTACGACGTGCCCGACTACGCCGGCAAGATCGTGAATAACTGGCTGAAGC
46	nsp3STOP_XbaI_rvs	AAATCTAGATTATCAGCCGCCCTTCAGGGC
47	InactiveProt_P2_rvs	GCCAGGTAGGCATTGTTGTCG

### Transfection

HEK293T cells were seeded into 10-cm-diameter dishes at a density of 5 × 10^6^ cells per plate or on top of glass cover slips in 24-well plates at a density of 5 × 10^4^ cells per well. To improve adherence of the HEK293T cells, cover slips were incubated with Rat collagen (Sigma-Aldrich) for 1 hour at 37°C followed by washing with PBS before cell seeding. The day after seeding, the DMEM medium was replaced by fresh medium. The transfection reagent polyethyleneimine (PEI; Sigma-Aldrich) and plasmid DNA were diluted in reduced serum OPTIMEM media (ThermoFisher) in separate reaction tubes at a ratio of 3:1 corresponding to 1.5 µL PEI and 0.5 µg DNA per well. After vortexing for 10 s, the mix was incubated 18 min at RT. For IF or EM analysis, the cells were washed once with PBS and fixed with paraformaldehyde (PFA, 4%) or EM fixative (2.5% glutaraldehyde, 2% sucrose, 50 mM KCl, 2.6 mM MgCl_2_, 2.6 mM CaCl_2_ in 50 mM Caco buffer), respectively.

### Immunoprecipitation

All steps of the IP procedure were performed on ice and in the presence of protease inhibitors (cOmplete EDTA-free protease inhibitor cocktail, Sigma-Aldrich) to prevent protein degradation. For IP under membrane-solubilizing conditions, HEK293T cells were transfected as described, washed 24 hours post-transfection (hpt), and lysed by the addition of 1 mL of membrane-solubilizing lysis buffer [50 mM Tris-HCl, 150 mM NaCl, 1 mM EDTA, 0.5% dodecylmaltoside (ThermoFisher Scientific), 5% glycerol] per 10 cm dish. After incubation for 10 min on ice with regular inverting, samples were centrifuged at 15,000 × *g* for 15 min at 4°C. The supernatant was transferred into a fresh tube and protein concentration measured by Bradford assay (BioRad). Protein amounts were equalized to 1 mg total protein as input and the volume equalized before mixing with 50 µL µMACS anti-HA beads (Miltenyi Biotec). Magnetic columns (Miltenyi Biotec) were inserted in the magnetic stand and pre-washed with 300 µL of lysis buffer. After incubation under constant rotation for 1.5 hours at 4°C, the mix was added on top of the column and the column washed with 3 mL of lysis buffer before incubation with 25 µL elution buffer (3% SDS in 50 mM Tris-HCl pH 7.4). After 5 min, an additional 75 µL of elution buffer was added and the elution collected. After detergent removal by acetone precipitation, samples were resuspended in 40 µL urea/thiourea buffer (6 M urea, 2 M thiourea in 10 mM HEPES, pH 8.0), and cysteine residues were reduced and alkylated with dithiothreitol and iodoacetamide as previously described (58). Protein digestion was performed by subsequent addition of 1 µg LysC (3 hours, 25°C; FUJIFILM Wako Pure Chemical Corporation, Richmond, VA, USA) and 1 µg trypsin (Promega, Walldorf, Germany) in 160 µL digestion buffer (50 mM ammonium bicarbonate, pH 8.0) at 25°C overnight. Peptides were desalted and concentrated using C18 Stage-Tips (59) and processed for LC-MS/MS. For the validation of interaction partners, 15% of the input and 15% of the IP were aliquoted, mixed with sample buffer, boiled, and analyzed by western blot. Specific primary and HRP-conjugated secondary antibodies were used to detect proteins. GAPDH served as a loading control.

### Liquid chromatography and trapped ion mobility spectrometry quadrupole time-of-flight settings

Samples were analyzed on a nanoElute (plug-in v.1.1.0.27; Bruker) coupled to a trapped ion mobility spectrometry quadrupole time of flight (timsTOF Pro) (Bruker) equipped with a CaptiveSpray source. Peptides were injected into a Trap cartridge (5 mm × 300 µm, 5 µm C18; Thermo Fisher Scientific) and separated on a 25 cm × 75 µm analytical column, 1.6 µm C18 beads with a packed emitter tip (IonOpticks) as previously described (58). The timsTOF Pro was operated in parallel accumulation-serial fragmentation (PASEF) mode using Compass Hystar v.5.0.36.0 and the following settings: mass range 100–1,700 m/z, 1/K0 start 0.6 V⋅s/cm^2^ End 1.6 V⋅s/cm^2^; ramp time 110.1 ms; lock duty cycle to 100%; capillary voltage 1,600 V; dry gas 3 L/min; dry temperature 180°C . The PASEF settings were 10 tandem mass spectrometry scans (total cycle time, 1.27 s); charge range 0–5; active exclusion for 0.4 min; scheduling target intensity 10,000; intensity threshold 2,500; collision-induced dissociation energy 42 eV.

### Raw mass spectrometry data processing and analysis

Raw MS data were processed with the MaxQuant software v.1.6.17 using the built-in Andromeda search engine to search against the human proteome (UniprotKB, release 2019_10) containing forward and reverse sequences concatenated with the SARS-CoV-2 polyprotein with the individual viral open-reading frames manually annotated and the label-free quantitation algorithm (60). Additionally, the intensity-based absolute quantification (iBAQ) algorithm and match between runs option were used. In MaxQuant, carbamidomethylation was set as fixed and methionine oxidation and N-acetylation as variable modifications. Search peptide tolerance was set at 70 p.p.m., and the main search was set at 30 p.p.m. Experiment type was set as TIMS-data-dependent acquisition with no modification to the default settings. Search results were filtered with a false discovery rate of 0.01 for peptide and protein identification. The Perseus software v.1.6.10.43 was used to process the data further (61). Protein tables were filtered to eliminate the identifications from the reverse database and common contaminants. When analyzing the MS data, only proteins identified on the basis of at least one peptide and a minimum of three quantitation events in at least one experimental group were considered. The iBAQ protein intensity values were log-transformed; missing values were filled by imputation with random numbers drawn from a normal distribution calculated for each sample, and principal component analysis was used to identify and remove outliers. In total, one biological replicate was removed from each experimental condition with the exception of the pcDNA3.1 group. The mass spectrometry-based proteomics data have been deposited at the ProteomeXchange Consortium (http://proteomecentral.proteomexchange.org) via the PRIDE partner repository (PXD036206). Source data are provided with this paper (Table S1).

### Western blot analysis

Western blotting was performed by loading the samples on 8% or 10% polyacrylamide gels. Proteins were transferred to polyvinylidene fluoride membranes by wet blotting at 100 mA and 4°C overnight. The next day, membranes were blocked with skim milk (5%) in PBS-Tween (0.2%) for 1 hour. After washing, the membranes were incubated with the primary antibody (Table 2) diluted in PBS-Tween (0.2%) as indicated for 2 hours at RT or overnight at 4°C. After three washes with PBS-Tween (0.2%) for 15 min each, the membranes were incubated with secondary antibody conjugated to horseradish peroxidase diluted in PBS-Tween (0.2%) for 45 min at RT. Peroxidase signal was detected with enhanced chemiluminescence solution (Perkin Elmer, Waltham, MA, USA) using the ChemoCam 6.0 ECL system (Intas Imaging, Goettingen, Germany).

**TABLE 2:** Antibodies

Antibody	Western blot	IF	Manufacturer	Identifier
Mouse anti-GAPDH	1:1,000		Santa Cruz Biotechnology	Cat.#sc365062
Rabbit anti-nsp3		1:200	Abcam	Cat.#ab181620
Rabbit anti-nsp3	1:500		Genetex	Cat.#GTX135614
Mouse IgG1 anti-HA	1:1,000	1:500	Sigma Aldrich	Cat.#H3663
Rabbit anti-HA		1:500	Enzo Life Sciences	Cat.# ABS203
Rabbit anti-HA		1:500	Invitrogen	Cat.#PA1-985
Mouse IgG2a anti-V5	1:1,000	1:500	ThermoFisher Scientific	Cat.#R960-25
Rabbit anti-PDI		1:100	Sigma Aldrich	Cat.#P7372
Mouse anti-RTN3		1:100	Santa Cruz Biotechnology	Cat.#sc-374599
Rabbit anti-PMP70		1:100	Abcam	Cat.#ab188499
Rabbit anti-GM130		1:100	Cell Signaling Technology	Cat.#12480
Mouse anti-ATP5B	1:500	1:100	Abcam	Cat.#ab14730
Mouse anti-FMR1	1:500		ThermoFisher Scientific	Cat.#TA504290
Rabbit anti-FXR1	1:500		Sino Biological	Cat.#105112-T42
Rabbit anti-TMEM33	1:500		ThermoFisher Scientific	Cat.#A305-587A
Rabbit anti-FXR2	1:500		Cell Signaling Technology	Cat.#7098
Rabbit anti-MKRN2	1:1,000		ThermoFisher Scientific	Cat.#PA5-58004
Rabbit anti-HSD17B12	1:500		ThermoFisher Scientific	Cat.#PA5-112959
Goat anti-mouse IgG-HRP	1:10,000		Sigma-Aldrich	Cat.#A4416
Goat anti-rabbit IgG-HRP	1:5,000		Sigma-Aldrich	Cat.#A6154
Alexa Fluor 488 donkey anti-mouse IgG		1:1,000	ThermoFisher Scientific	Cat.#A-21202
Alexa Fluor 488 donkey anti-mouse IgG2a		1:1,000	ThermoFisher Scientific	Cat.#A-21131
Alexa Fluor 568 donkey anti-mouse IgG		1:1,000	ThermoFisher Scientific	Cat.#A-10037
Alexa Fluor 647 donkey anti-mouse IgG		1:1,000	ThermoFisher Scientific	Cat.#A-31571
Alexa Fluor 647 goat anti-mouse IgG1		1:1,000	ThermoFisher Scientific	Cat.#A-21240
Alexa Fluor 488 donkey anti-rabbit IgG		1:1,000	ThermoFisher Scientific	Cat.#A-21206
Alexa Fluor 568 donkey anti-rabbit		1:1,000	ThermoFisher Scientific	Cat.#A-10042
Alexa Fluor 647 donkey anti-rabbit		1:1,000	ThermoFisher Scientific	Cat.#A_31573

### Indirect immunofluorescence

Cells were transfected and fixed 24 hours post-transfection with PFA (4%). After washing with PBS, coverslips were incubated with Triton-X100 (0.2%) in PBS for 15 min at RT for permeabilization before blocking with skim milk (1%) in PBS-Tween (0.01%) for 1 hour. Coverslips were inverted and placed on 40 µL of pre-diluted primary antibody for 1.5 hours of incubation or overnight at 4°C. Coverslips were washed three times with PBS-Tween (0.02%) for 15 min. Fluorescently labeled secondary antibodies were diluted as indicated, coverslips inverted and placed on 40 µL of diluted secondary antibody for incubation for 1 hour in the dark. Finally, coverslips were washed three times for 15 min with PBS-Tween (0.02%) and mounted on glass slides using DAPI Fluoromount G (Southern Biotech).

### Transmission electron microscopy and electron tomography

Cells were washed and fixed 18 or 24 hpt in EM fixative [2.5% glutaraldehyde in 50 mM cacodylate buffer (pH 7.2) containing 2.6 mM MgCl_2_, 2.6 mM CaCl_2_, 50 mM KCl, and 2% sucrose] for 30 min at RT. Fixed cells were washed five times with 50 mM cacodylate buffer for 5 min before staining with osmium tetroxide (2%) in 50 mM cacodylate buffer for 40 min on ice. The staining was removed by three washes with water and cells stored at 4°C overnight. The next day, the cells were incubated with uranyl acetate (0.5%) for 30 min. Cells were dehydrated with an ethanol dilution series at an increment of 10% starting at 40% ethanol and going to 100% ethanol in water with 5 min for each step. The last two steps were performed at an increment of 5% (95% and 100%) and repeated twice for 10 min each. Cells were then embedded in epoxy resin mix (Araldite 502/Embed 812 kit, Electron Microscopy Series) and incubated at 60°C for at least 2 days to allow complete polymerization. Glass coverslips were removed by cold shock using liquid nitrogen, and samples cut into sections of 70 nM with a diamond knife (Diatome) in an Ultracut UCT microtome (Leica). Samples for electron tomography were cut into 250 nM sections and processed as described previously (62). For the EM-based host factor screen, an overview image of the grid hexagons was taken at 200× magnification using SerialEM (63). Micrographs were then acquired from all cells within the hexagon at a 10,000× magnification, and the percentage of cells harboring DMVs quantified. Additionally, the area occupied by DMVs in all imaged cells was quantified, and the diameter of individual DMVs was determined using Fiji for at least 50 DMVs per replicate and a total of at least 300 DMVs per condition.

### Reporter cell line siRNA screen

Pools of three small-interfering RNAs targeting the different interaction partners, a non-targeting control siRNA as well as a pool targeting the positive control ACE2 were acquired from Dharmacon. The siRNA pool (25 nM) was mixed with OptiMEM containing the transfection reagent Lipofectamine RNAi MAX (ThermoFisher). The mix was incubated 18 min at RT before addition of 6,000 A549-ACE2-RC cells per well of a 96-well black wall glass microscopy plate (ThermoFisher). And 48 hours post-transfection, cells were infected with SARS-CoV-2 (MOI = 5) for 24 hours before fixation with formaldehyde. Cells were stained with Hoechst (1:3,000) for 20 min at RT and subsequently analyzed with a Zeiss CellDiscoverer 7 widefield fluorescence microscope. A semi-automated image analysis pipeline based on CellProfiler and CellProfiler analyst was used to quantify the percentage of infected cells.

### Quantitative real-time PCR

Total RNA from cells was harvested and isolated using NucleoSpin RNAmini Kit (Macherey Nagel), according to the manufacturer’s protocol. cDNA was synthesized using the high-capacity cDNA Reverse Transcription Kit (Applied Biosystems), according to the manufacturer’s protocol. cDNA samples were diluted 1:20 in RNAse/DNAse-free H_2_O. qRT-PCR analysis was performed on a CFX96 Real-Time System (BioRad) using target-specific primers and the iTaq Universal SYBR green mastermix (Bio-Rad). Ct values of each sample were averaged across three technical replicates and then normalized to the transcript levels of the housekeeping control GAPDH. Fold change values were calculated by normalizing to the non-targeting control and used as a measure of gene expression.

### Bioinformatic analysis

Images were analyzed with the Fiji image analysis software (64). EM images were acquired with SerialEM and analyzed with the IMOD software package (63, 65, 66). ET images were aligned in IMOD with the etomo plugin, semi-automatically segmented in Ilastik and tomographic models, rendered using the blender software package (67, 68). Statistical analysis and graph generation were performed using GraphPad prism software package. Figures and schemes were designed in Adobe Illustrator utilizing graphics from bioicons by Hanna Vega and Marcel Tisch. String network analysis and gene ontology analysis were performed using STRING v11.0 (69).

## SUPPLEMENTAL MATERIAL

Supplemental table legend jvi.00878-23-s0001.docx Legend of Table S1.

Table S1 jvi.00878-23-s0002.xlsx Proteomics dataset.

ASM does not own the copyrights to Supplemental Material that may be linked to, or accessed through, an article. The authors have granted ASM a non-exclusive, world-wide license to publish the Supplemental Material files. Please contact the corresponding author directly for reuse.
