# Supplementary material for: Identification of host dependency factors involved in SARS-CoV-2 replication organelle formation through proteomics and ultrastructural analysis
Source: J Virol. 2023 Oct 31;97(11):e00878-23. doi: 10.1128/jvi.00878-23 (PMC10688318; doi:10.1128/jvi.00878-23)
Supplement: Supplemental table legend — Legend of Table S1. [file jvi.00878-23-s0001.docx]

**Legend of Supplemental Table S1**

**Table S1: Mass-spectrometry data of the SARS-CoV-2 nsp3/4 interactome.**

(A) List of all identified proteins in each experimental group by AP-LC-MS/MS, including iBAQ intensities and the outcome of the statistical analysis. (B) Selection of prioritized SARS-CoV-2 nsp3/4 interacting proteins for validation by RNAi.
